# Aberrant brain responses to emotionally valent words is normalised after cognitive behavioural therapy in female depressed adolescents

**DOI:** 10.1016/j.jad.2015.09.008

**Published:** 2016-01-01

**Authors:** Jie-Yu Chuang, Kirstie J Whitaker, Graham K Murray, Rebecca Elliott, Cindy C Hagan, Julia ME Graham, Cinly Ooi, Roger Tait, Rosemary J Holt, Adrienne O van Nieuwenhuizen, Shirley Reynolds, Paul O Wilkinson, Edward T Bullmore, Belinda R Lennox, Barbara J Sahakian, Ian Goodyer, John Suckling

**Affiliations:** a Department of Psychiatry, University of Cambridge; b Behavioural and Clinical Neuroscience Institute, University of Cambridge; c Cambridgeshire and Peterborough NHS Foundation Trust; d Institute of Brain, Behaviour and Mental Health, University of Manchester; e Department of Psychology, Columbia University; f University of Cambridge; g School of psychology and Clinical language Sciences, University of Reading; h Department of Psychiatry, University of Oxford

**Keywords:** Adolescent depression, Functional magnetic resonance imaging, Cognitive behaviour therapy, Positive stimuli

## Abstract

**Background:**

Depression in adolescence is debilitating with high recurrence in adulthood, yet its pathophysiological mechanism remains enigmatic. To examine the interaction between emotion, cognition and treatment, functional brain responses to sad and happy distractors in an affective go/no-go task were explored before and after Cognitive Behavioural Therapy (CBT) in depressed female adolescents, and healthy participants.

**Methods:**

Eighty-two Depressed and 24 healthy female adolescents, aged 12–17 years, performed a functional magnetic resonance imaging (fMRI) affective go/no-go task at baseline. Participants were instructed to withhold their responses upon seeing happy or sad words. Among these participants, 13 patients had CBT over approximately 30 weeks. These participants and 20 matched controls then repeated the task.

**Results:**

At baseline, increased activation in response to happy relative to neutral distractors was observed in the orbitofrontal cortex in depressed patients which was normalised after CBT. No significant group differences were found behaviourally or in brain activation in response to sad distractors. Improvements in symptoms (mean: 9.31, 95% CI: 5.35–13.27) were related at trend-level to activation changes in orbitofrontal cortex.

**Limitations:**

In the follow-up section, a limited number of post-CBT patients were recruited.

**Conclusions:**

To our knowledge, this is the first fMRI study addressing the effect of CBT in adolescent depression. Although a bias toward negative information is widely accepted as a hallmark of depression, aberrant brain hyperactivity to positive distractors was found and normalised after CBT. Research, assessment and treatment focused on positive stimuli could be a future consideration. Moreover, a pathophysiological mechanism distinct from adult depression may be suggested and awaits further exploration.

## Highlights

• No bias toward negative information was found in female depressed adolescents.
• Abnormal hyperactivity to positive distractors was found in the orbitofrontal cortex.
• This aberrant brain activity was normalized after cognitive behavioural therapy.
• A mechanism distinct from adult depression may be suggested in adolescent depression.

## 1 Introduction

Adolescence is turbulent with an imbalance in brain maturation between subcortical and prefrontal areas (Casey and Caudle, 2013). Major depressive disorder (MDD) during adolescence is associated with severe functional impairment, suicide, and high recurrence rate in adulthood (Thapar et al., 2012). The pathophysiology of adolescent depression may be elucidated by investigation of the neural correlates. However, in contrast with adult depression, only a few neuroimaging studies have been conducted to date, and no meta-analyses providing a convergent description in adolescents are currently available (Hagan et al., 2013).

In everyday life, most adults are able to suppress responses to neutral or emotional distractions to achieve goals. By contrast, adolescence is associated with heightened reactivity and poor self control when facing positive or negative emotional cues (Casey, 2014). This may be enhanced in depressed adolescents who respond more impulsively when faced with an emotionally valent distractor in a inhibitory control task (Maalouf et al., 2012). In fact, even with neutral stimuli, aberrant response to distractors has been associated with suicidality in adolescent depression (Pan et al., 2011). Indeed, attentional bias towards emotional stimuli has been postulated to be a significant component in the aetiology and maintenance of depression (Epp et al., 2012) with a resulting disturbance to daily performance.

A common task exploring attentional bias, the affective go/no-go (AGNG) task requires participants to respond (e.g. press a button) to target (‘go’) stimuli that are emotionally valent (e.g. sad) whilst inhibiting their response to distractor (‘no-go’) stimuli of different valence (e.g. happy). From a functional imaging perspective, activation is increased in healthy adults in lateral inferior prefrontal cortex in response to positive versus neutral distractors, and in anterior cingulate, insula and hippocampus in response to negative versus neutral distractors (Van Holst et al., 2012). Compared with healthy adults, depressed adults show increased activation in the right lateral orbitofrontal cortex and bilateral anterior temporal cortex in response to sad versus neutral distractors (Elliott et al., 2002) which seemingly indicates a bias toward negative information. However, the ability of positive stimuli to induce aberrant activations has also been demonstrated in a depression meta-analysis (Groenewold et al., 2013). Indeed, despite a general belief that mood-congruent stimuli should be more salient in depression (Epp et al., 2012), the supporting evidence from affective neuroscience is not conclusive. First, a fMRI meta-analysis of emotional tasks in adults with depression has demonstrated that both positive and negative stimuli induce extended and overlapping abnormal activations (Groenewold et al., 2013). More specific to attentional bias, a further meta-analysis reveals the ability of positive stimuli to exert large stroop-like effects on depressed patients (Epp et al., 2012). Furthermore, a review of the literature indicates that numerous studies failed to demonstrate attentional bias towards negative information in depression (Elliott et al., 2011). Rather, there is the suggestion of a general, rather than emotion-congruent attentional bias, or argument for biases in more effortful processing such as interpretation and memory instead of attention (Epp et al., 2012). However, there is also a debate on the general difficulty in conflict monitoring or inhibition (Epp et al., 2012). It has even been suggested that a shared reaction to threat is perceived regardless of valence in these tasks (Epp et al., 2012). Different tasks and heterogeneity in patients may also contribute to the inconsistent findings (Elliott et al., 2011). Consequently, we investigated the responses to both sad versus neutral and happy versus neutral distractors in adolescent depression.

UK National Institute for Health and Care Excellence guidelines for the initial clinical management of moderate to severe MDD in adolescence recommends that a psychological therapy be offered or combined with a selective serotonin uptake inhibitor (SSRI, specifically fluoxetine) (Hopkins et al., 2015). CBT is arguably the most commonly used psychotherapy with several neuroimaging studies conducted in adults. In addition to mutual modulation of several cortico-limbic regions, CBT is associated with changes in prefrontal regions more than subcortical structures, which are likely to be regulated by antidepressants (Kennedy et al., 2007). CBT treatment effects in adult depression have been demonstrated in ventromedial prefrontal cortex (Ritchey et al., 2011), medial prefrontal cortex (Kennedy et al., 2007; Yoshimura et al., 2013), occipital-temporal cortex (Kennedy et al., 2007), orbitofrontal cortex (Kennedy et al., 2007), ventral (Yoshimura et al., 2013) and dorsal anterior cingulate (Kennedy et al., 2007). Aberrancy in response to emotional stimuli has been shown to be diminished in the medial prefrontal cortex (Ritchey et al., 2011; Yoshimura et al., 2013) after CBT. However, despite many similarities, there are prominent differences in treatment responses between adult and adolescent depressed patients; for instance, tricyclic antidepressants are effective only in the former, and SSRI antidepressants are more likely to induce suicidal thoughts in the latter (Thapar et al., 2012). Indeed, the neurophysiological mechanism of CBT in adolescent depression awaits exploration.

We hypothesise that aberrant responses towards happy and sad distractors in the fMRI AGNG task would be observed at baseline and later corrected after CBT in depressed female adolescents. To our knowledge, no previous study has examined brain activation both pre- and post-CBT in depressed adolescents, thus no specific brain region was predetermined as differing between patients and controls. Additionally, gender difference is a long-debated heterogeneity issue with depressed males showing higher ratings of anhedonia (Bennett et al., 2005) and lower tendency to ruminate (Johnson and Whisman, 2013). Consequently, we restricted our analysis to females.

## 2 Methods

### 2.1 Participants

Patients were recruited from the Improving Mood with Psychoanalytic and Cognitive Therapies (IMPACT), a pragmatic, effectiveness randomised clinical trial (Goodyer et al., 2011) in East Anglia, North London and North West of England, which is designed to determine the efficacy of psychotherapy. A sub-sample of patients from East Anglia and North London were invited to participate in an adjunctive study, MR-IMPACT, aimed at exploring the pathophysiology of depression using MRI(Hagan et al., 2013). Healthy female controls matched for age, intelligence quotient and handedness were also recruited in the MR-IMPACT study(Hagan et al., 2013).

The data in this analysis were obtained from a longitudinal assessment of patients and controls recruited into MR-IMPACT. All participants underwent baseline assessment. Those IMPACT participants randomized to CBT therapy (Supplementary material 1) and completed multiple CBT sessions (12.85±4.49, range: 5–21 sessions within 243.15±49.81 days) underwent follow-up assessment (Hagan et al., 2013). Control participants were also invited to return for a follow-up assessment. 94 patients and 29 controls were initially recruited with 12 patients and 5 controls excluded due to ineligibility (Supplementary material 2). The exclusion rates between patient and control groups were similar (12/94 and 5/29). Baseline data presented are from 82 depressed and 24 healthy female adolescents (Supplementary material 2) (Table 1): termed the *full group*. Among them, 13 patients and 20 controls returned for follow-up assessment (Table 2), and are termed the *follow-up group*. Demographic data and behavioural measures were compared between the *follow-up group* and the *full group*; the *follow-up group* and those who were not followed up in the *full group* (*full group* minus *follow-up group*).

### 2.2 Affective go/no-go task (AGNG)

Happy, sad and neutral words were presented to participants during MRI data acquisition using a block paradigm task design (Elliott et al., 2002). Words did not differ in terms of length or usage frequency Hofland K (1982). There were 7 types of blocks each repeating 3 times, consisting of randomly ordered targets/distractors (10 of each type): sad/neutral (SN), sad/happy (SH), neutral/sad (NS), neutral/happy (NH), happy/sad (HS), happy/neutral (HN) and neutral italic/plain (IP). Equal numbers of happy and sad words were used with mood induction unlikely to occur. The inter-block interval was 12 seconds with the first 4 seconds of each block used to present the instructions for that block. Each word was presented for 450 ms with a 750 ms inter-stimulus interval (ITI) (Hagan et al., 2013). Participants were asked to press a button with their right index finger when presented with a target word (‘go’), and to inhibit responses to distractor words (‘no-go’). All participants completed a go/no-go practice task (living versus non-living stimuli) prior to scanning.

### 2.3 Behavioural data analysis

The following analyses were based on two contrasts: “the happy distractor contrast” (SH-SN) and “the sad distractor contrast” (HS-HN) with targets fixed and distractors differing in valence in each contrast. Behavioural measures tailored to the “happy distractor contrast” and “sad distractor contrast” were recorded with three variables: mean reaction time of correct go, incorrect go (omission error), and incorrect no-go (commission error). The timing of responses was measured when participants’ released the button. Analysis of covariance (ANCOVA) was performed in SPSS (version 21) at baseline on the behavioural measures of the *full group*, with group (depressed or control) as a factor, and age at baseline as a covariate. In the *follow-up group*, a similar ANCOVA model with group (depressed or control) and time (baseline or follow-up) as factors was assessed for the behavioural measures, testing the group x time interaction.

### 2.4 fMRI analysis overview

FMRI data were acquired and preprocessed (Supplementary material 3). Firstly, mean activations and deactivations of the “happy distractor contrast” and the “sad distractor contrast” were determined in the *full group* (from both depressed participants and controls). Secondly, restricting the analysis to these mean activated and deactivated regions, we identified regions showing differential between-group response in the *follow-up group*. Finally, restricting the analysis to any identified regions, effects of CBT treatment were investigated via exploration of group x time interactions in the *follow-up group*.

### 2.5 Between-subject fMRI data analysis

All of the following neuroimaging analyses were performed with FEAT. Statistic images were thresholded using clusters generated by Z>2.3 and a cluster significance threshold of P=0.05 family-wise error corrected (Worsley, 2001). Firstly, following initial single-subject first level analysis to generate parametric maps for the “happy distractor contrast” and the “sad distractor contrast”, all maps were normalized to standard Montreal Neurological Institute (MNI) space (Worsley, 2001). Secondly, a whole-brain one-sample T test controlling for baseline age, determined significant mean activation and deactivation patterns of the two contrasts in the baseline data of the *full group*. Thirdly, restricting the analysis to these mean activated or deactivated regions, a two-sample T test controlling for baseline age was performed to identify areas of between-group difference in the baseline data of the *follow-up group*. Finally, we extracted mean percent signal changes from regions of between-group difference in both the baseline and follow-up fMRI data of the *follow-up group*. An ANCOVA was then performed with the temporal difference of these percent signal changes as the dependant variable, group as the independent variable, and baseline ages as a covariate.

### 2.6 Relationship of fMRI to symptoms

Symptoms were assessed via the scoring of Short Mood and Feelings Questionnaire (SMFQ). To explore the association between changes in functional imaging and symptom change in the *follow-up group*, we initially extracted baseline and follow-up mean percent signal changes from the regions of between-group difference identified with baseline data in the prior analysis. First, the partial correlation covarying for baseline age was tested between the baseline functionally imaging response and the baseline depressive symptoms. Then, the partial correlation covarying for baseline age, was again tested, but between the difference of mean percent signal change at baseline versus follow-up, and the difference in symptom score, normalized by the baseline symptom score.

## 3 Results

### 3.1 Demographic results

Patients were more anxious and depressed than controls but they did not differ significantly in terms of age, IQ, handedness, and duration between baseline and follow-up (Table 1 and Table 2). Similarly, these demographic data did not differ significantly (except the depressed patients in the *follow-up group* had higher scores of trait anxiety than those patients without follow-up) between the *follow-up group* and *those who were not followed up* (*full group* minus *follow-up group*) (Supplementary Table 1). In the *full group*, there were 9 left-handed patients and 2 left-handed controls. However, handedness scores did not differ significantly between patients and controls in either the *full group* or the *follow-up group* (Table 1 and Table 2).

### 3.2 Behavioural results

The baseline behavioural data did not differ significantly between the *follow-up group* and *those who were not followed up* (*full group* minus *follow-up group*) (Supplementary Table 1). There were no significant baseline group differences compared to controls in any behavioural measures of the *full group.* In the *follow-up group*, there was a significant improvement in SMFQ after CBT (mean=9.31, 95% CI: 5.35–13.27, *t*(12)=5.12, *p*=2.52*10^−4^) in the depressed adolescents, although no significant group x time interactions of the behavioural measures were identified (Supplementary Table 2).

### 3.3 Between-group differences in brain activation at baseline

**Fig. 1:**
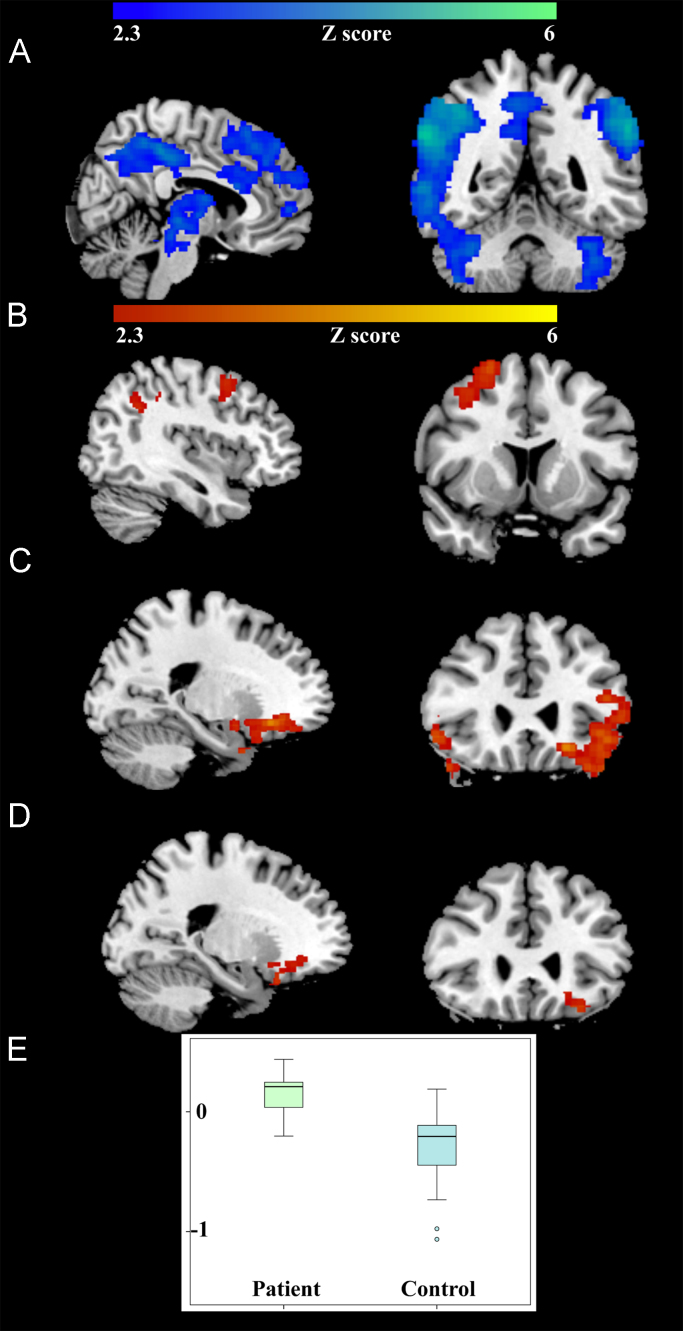
Significant brain activation at baseline. Mean activations of the *full group* associated with the “sad distractor contrast” were located in anterior cingulate, cerebellum and insula (A). Mean activations of the “happy distractor contrast” were located in superior frontal gyrus and supramarginal gyrus (B). Mean activations of the reverse of “happy distractor contrast” were found in dorsolateral prefrontal cortex, temporal cortex, and orbitofrontal cortex (C). With analysis restricted to the region of mean activation of the “happy distractor contrast”, significant between-group differences were located in the orbitofrontal cortex (D) of the *follow-up group*. In this orbitofrontal region (D), baseline percent signal change was extracted with the patient group showing significantly higher (t(31)=4.86 *p*=3.2*10^−5^) value than the control group (E).

With the analysis restricted to the mean activation and deactivation maps, in the *follow-up group* there was a significant increase in activation in orbitofrontal cortex in depressed patients’ response to “happy distractor contrast” (Fig. 1, Table 3). No significant group difference was found with the “sad distractor contrast”.

### 3.4 Changes in brain activation associated with CBT treatment

**Fig. 2:**
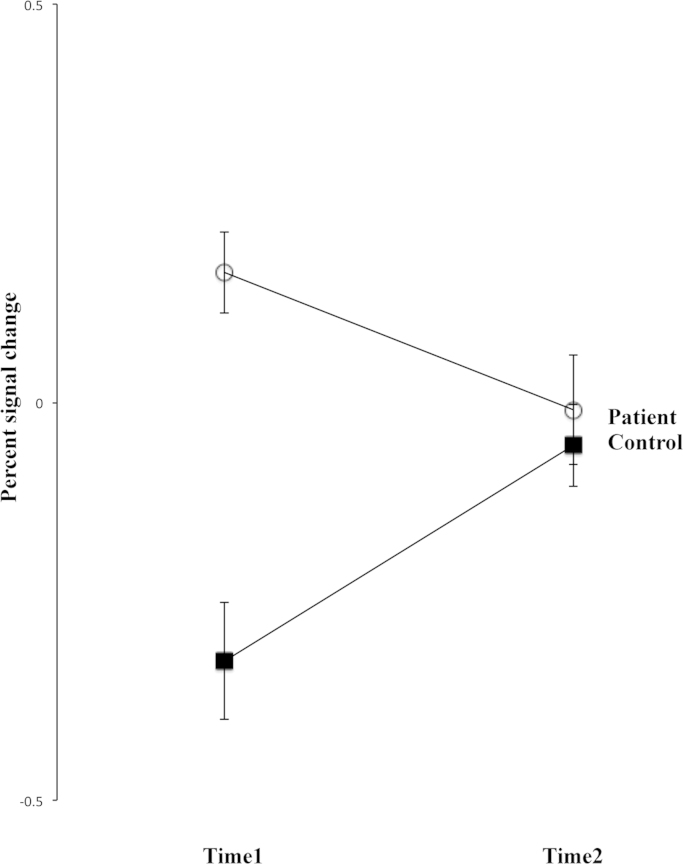
Group x time interaction in the orbitofrontal cortex. There was a significant between-group difference of the mean percent signal change of the “happy distractor contrast” in the baseline data of the *follow-up group*. However, this difference was non-significant after CBT treatment in the follow-up assessment. Furthermore, the temporal changes of the mean percent signal change in both patient and control groups were significant. Standard errors of the mean are shown.

Within this orbitofrontal region, the group x time interaction of the percent signal changes was explored. Residuals of ANCOVA were not normally distributed, and thus the dependent variable of temporal difference, x, of the percent signal change was transformed by the function: log(x+3), which resulted in normally distributed residuals. There was a significant group x time interaction (F(1, 30)=9.12, *p*=5.12*10^−3^) (Fig. 2) with post-hoc testing identifying significant temporal changes in both patient (*t*(30)=28.74, *p*<1.00*10^−5^) and control group (*t*(30)=40.49, *p*<1.00*10^−5^), but in opposing directions of effect with decreases in patients and increases in controls. The group x time interaction remained significant after adding the time interval between scans as a covariate (F(1, 29)=9.00, *p*=5.51*10^−3^). There was a significant group effect at baseline (F(1, 30)=22.60, *p*<1.00*10^−4^) which was diminished at follow-up (F(1, 30)=0.34, *p*=0.56).

### 3.5 Relationship of brain activation and symptoms

**Fig. 3:**
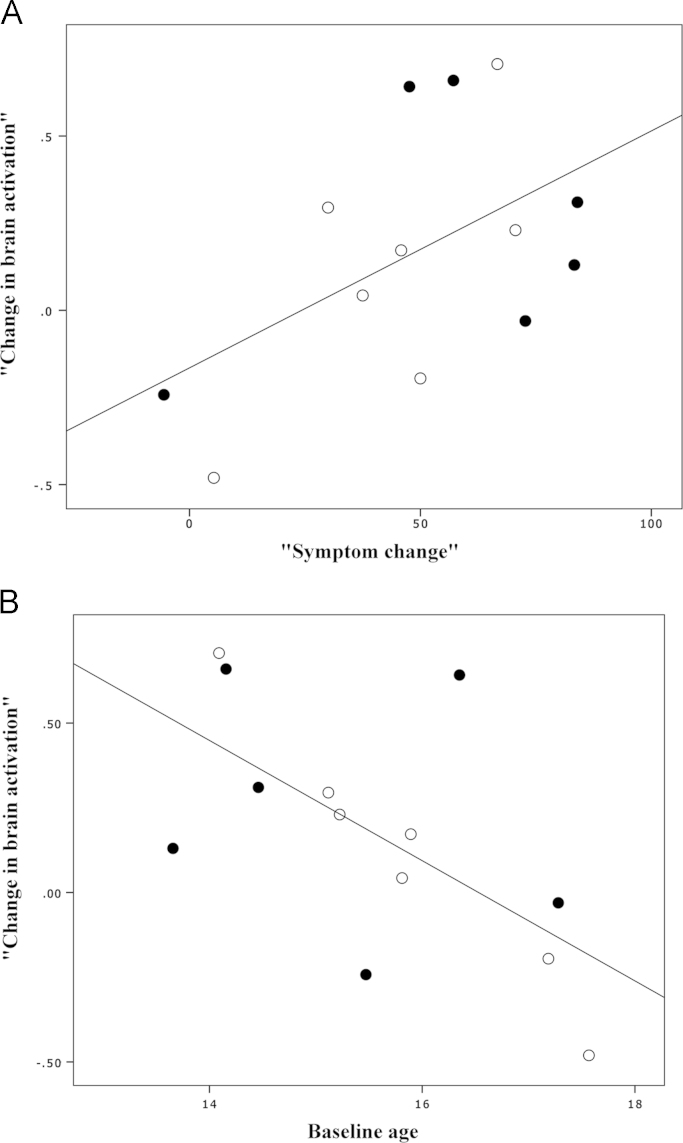
Relationship of changes in brain activation and symptoms in depressed patients of the *follow-up group*. “Change in brain activation” is baseline minus follow-up mean percent signal change within the orbitofrontal region identified as having a significant between-group difference. “Symptom change” is the baseline minus follow-up SMFQ score divided by the baseline score. (A) When baseline age was not controlled, there was a trend-level correlation between brain activation and symptom changes; *R*=0.52, *p*=0.07. (B) There was a significant correlation between baseline age and change in brain activation; *R*=−0.63, *p*=0.02. Depressed participants taking medication are shown with solid circles.

For the “happy distractor contrast” in the patients of the *follow-up group*, there was a non-significant relationship between baseline brain activity and concurrent SMFQ score (R=−0.03, *p*=0.77). There was a moderate correlation of temporal difference of mean percent signal change in the orbitofrontal cortex with the temporal SMFQ change normalized by baseline SMFQ score (*R*=0.35, *p*=0.26). When baseline age was not controlled, the correlation was at trend level (*R*=0.52, *p*=0.07). Also, baseline age was found to be significantly correlated with the temporal percent signal change (*R*=−0.63, *p*=0.02), and moderately correlated with the temporal SMFQ change normalized by baseline SMFQ score (*R*=−0.44, *p*=0.14) (Fig. 3). In other words, older patients tended to have relatively reduced fMRI BOLD signal and symptom changes. Furthermore, combining antidepressants with CBT did not appear to exert an additive treatment effect (Fig. 3).

## 4 Discussion

Using an affective go/no-go task, aberrant brain activation towards happy distractors was found and later corrected following CBT in the orbitofrontal cortex of depressed female adolescents.

### 4.1 The happy distractor contrast

We were able to demonstrate aberrant activations towards happy rather than sad stimuli in depressed female adolsecents. This finding echoes the inconsistent emotional bias literature, and further emphasise the importance of positive stimuli in depression. Accompanied by depressed mood, diminished pleasure has already been listed as one of the two essential mood criteria for major depressive disorder in the Diagnostic and Statistical Manual of Mental Disorders (DSM)-5. However, positive stimuli have not received equal attention in comparison to negative stimuli both in clinics and research. As a result, research focusing on positive stimuli, or diagnostic tools centred on assessment of positive affect are of potential interest.

Emotional processing deficits found in vulnerable, but well adolescents on an affective go/no-go task may act as a diagnostic biomarker for depressive disorders (Owens et al., 2012). Several studies have also reported persistent negative biases in remitted depressed adult patients (Elliott et al., 2011). However, there is no consensus on whether emotional bias is a state or trait feature of depression. We demonstrate that emotional bias of the brain activation might be reversible in adolescent depression.

### 4.2 The orbitofrontal cortex

As a key element of the reward system (Forbes and Dahl, 2005), the orbitofrontal cortex receives information from all sensory modalities, extensively connects with limbic system, and is responsible for decision processing (Wallis, 2007). Indeed, with substantial neural development in adolescence, the reward system might be vulnerable to dysregulation by depression during this period (Forbes and Dahl, 2005).

Disrupted connectivity of orbitofrontal cortex has been reported during processing of happy faces (Elliott et al., 2011). Furthermore, the most aberrant response to positive stimuli was found in orbitofrontal cortex in a meta-analysis of emotional tasks in adults with depression (Groenewold et al., 2013). Indeed, it has been proposed that the orbitofrontal cortex specifically modulates positive emotion (Groenewold et al., 2013). Taken together, abnormal activation of orbitofrontal cortex in our depressed female adolescents might be attributed to difficulty in modulating, and making decisions about responding to, or ignoring positive distractor stimuli.

### 4.3 Cognitive deficits

Review of the adult depression literature indicates an imbalance between emotion (ventromedial prefrontal cortex) and cognitive (dorsolateral prefrontal cortex) processing with negative emotion generation and reappraisal suggested as the underlying mechanisms, respectively (Koenigs and Grafman, 2009). However, a further qualitative review of the imaging data in adolescent depression suggests that orbitofrontal cortex instead of dorsolateral prefrontal cortex is among the most consistently reported aberrant regions (Kerestes et al., 2013).

Likewise, we did not demonstrate significant differences between healthy and depressed adolescents in performance or in activation of the dorsolateral prefrontal cortex (although this area was among the mean activation regions reacting to sad distractors) (Supplementary material 4). It has been proposed that orbitofrontal cortex has a central role in calculating the value of decisions, whereas the dorsolateral prefrontal cortex is responsible for using that value information to organise behaviours (Wallis, 2007). It is therefore possible that the progression of depression also follows this processing hierarchy, with functional changes demonstrated in the orbitofrontal cortex initially followed by dorsolateral prefrontal cortex impairment and associated difficulties with cognitive tasks at later stages of the disorder. Further studies with longer follow-up should be conducted.

### 4.4 Cognitive behavioural therapy

Components of CBT include cognitive restructuring, behavioural activation, and problem solving (Forbes and Dahl, 2005). Of all these components, behavioural activation is particularly related to positive stimuli via increasingly pleasurable activities (Forbes and Dahl, 2005). Due to relative cognitive immaturity of adolescents, behavioural modification was more emphasised in our study than typical adult CBT. Coincidently, critical deficits in adolescent female depression was targeted; i.e. response to positive stimuli. However, positive stimuli are not the main focus of current treatment for depression, and thus perhaps deserve more consideration in future development of therapy (Forbes and Dahl, 2005).

It has been reported that both antidepressant (Delaveau et al., 2011) and CBT (Kennedy et al., 2007) can alter the activation and metabolism of orbitofrontal cortex. Although limited participant numbers preclude statistical testing, our preliminary results suggested that combining antidepressant medication with CBT did not appear to exert an additive treatment effect (Fig. 3). Indeed, despite a higher improvement rate in adult depression, significant benefits of combined treatment over single CBT or antidepressant treatment have not been consistently found in adolescent depression (Dubicka et al., 2010). Furthermore, older depressed adolescents showed less symptomatic improvement and neuroimaging alterations possibly indicating less plasticity or distinct treatment mechanisms (Fig. 3). Of note, this prominent age effect might result in the non-significant correspondence between SMFQ and brain activation when age was controlled.

### 4.5 Limitations

A large number of participants were recruited for the baseline assessment whereas our *follow-up group* was relatively small, yet the number still outnumbered most brain imaging research addressing the effect of psychotherapy (Straub et al., 2015; Fujino et al., 2014). Although no significant behavioural difference was found, patients in our follow-up group had higher trait anxiety than patients without follow-up. Also, constrained by ethical considerations, we did not recruit another patient group without treatment. Consequently, we are unable to exclude natural course as a co-contributor to the temporal changes observed. Furthermore, adults were not included in our study. Future studies might consider tracing the trajectory of depression from adolescence to adulthood.

Significant group differences were found in brain responses, but not performance. This type of discrepancy has been frequently reported in the fMRI literature. Possible explanations include: higher sensitivity of brain signals than behavioural measures to detect group differences; or alternative neural strategies employed by patients to achieve comparable levels of performance (Murray et al., 2010). We were unable to differentiate which explanation best accounted for our data, nevertheless, the importance of aberrant brain activation cannot be overlooked. Although a good correlation between symptom alteration and brain activation change is desirable, only a trend-level correspondence was found. However, the majority of clinical scales were not designed with specific neural correlates in mind. Thus, it is possible that imaging findings only correspond to a limited number of components of the scales, resulting in reduced power to detect associations (Chuang et al., 2014).

Similar to several previous studies, healthy controls showed variability of signal change in repeated fMRI scans (Yoshimura et al., 2013). In our study, brain activation in response to happy distractors was increased from baseline to follow-up scan in healthy female adolescents. Possible causes for this temporal change include practice effects, developmental effects, and regression to the mean. We were unable to differentiate between these possibilities, nevertheless the direction of the change was opposite to that observed in the controls, and thus potentially associated with the CBT.

Restricted by the study design, we were only able to compare happy versus neutral distractors when controlled for sad targets; and sad versus neutral distractors when controlled for happy targets. Thus it will be interesting to see if the brain responses differ when targets of other valence are controlled (i.g. fearful). Verbal stimuli were used instead of faces or pictures, possibly resulting in reduced bottom-up limbic activation (Vercammen et al., 2012). Furthermore, considering the restricted attention span of adolescents, we chose not to prolong the task by separating emotional from cognitive processing in the study design. Future studies can, however, consider combining tasks with separate processing (such as go/no-go with eye-tracking (Epp et al., 2012)) or parametric designs (Schulz et al., 2009).

## 5 Conclusion

We were able to identify hyperactivity in orbitofrontal cortex in response to happy relative to neutral distractors in depressed female adolescents, with reversibility demonstrated after CBT. Reversibility of deficits and the importance of positive distractors in female adolescent depression were thus suggested. Research, assessment, and treatment focusing on positive stimuli are anticipated. Last but not least, the possible mechanistic difference between adult and adolescent depression should also be explored in future studies.

## Conflict of interest

Professor Sahakian reports personal fees from Cambridge Cognition, personal fees and other from Lundbeck, personal fees from Servier, grants from J&J Janssen, other from Otsuka, personal fees from Peak (Brainbow), outside the submitted work; Professor Bullmore works half-time for the Univeristy of Cambridge and half-time for GlaxoSmithKline. He holds stock in GlaxoSmithKline. Professor Goodyer reports personal fees from Lundbeck and holds grants from the Wellcome Trust and the Friends of Peterhouse Charity and NIHR-HTA. Dr. Wilkinson reports grants from MRC during the conduct of the study; personal fees from Lundbeck, personal fees from Takeda, outside the submitted work; and acts as a supervisor and trainer for interpersonal psychotherapy.

## Contributors

SR, ETB, BRL, BJS, IG, JS conceived and designed the experiment. CCH, JMEG, RJH, AON collected the data. JC, KW, GM, CO, RT analysed the data. JC, JS, RE drafted the manuscript. All authors critically reviewed the manuscript.

## Role of the funding source

The study was funded by the Medial Research Council (grant: G0802226). The IMPACT clinical trial was funded by the NHS Health Technology Assessment (HTA) Programme, Central Manchester and Manchester Children's University Hospitals NHS Trust, and the Cambridge and Peterborough Mental Health Trust. Additional support was provided by the jointly funded Medical Research Council/Wellcome Trust Behavioural and Clinical Neuroscience Institute, University of Cambridge, and the National Institute for Health Research (NIHR) Cambridge Biomedical Research Centre.

## Figures and Tables

**Table 1:** Demographic and baseline characteristics in the full group.

	Control (mean±SD)	Depression (mean±SD)	Between-group difference (*t* value/d.f.) (*p* value)
Number of subjects	24	82	–
Age (years)	15.89±1.42 (range:12.15–17.76)	15.72±1.10 (range:13.66–17.97)	−0.62/104
0.54
Estimated IQ	100.79±10.85 (range:82–120)	97.83±12.02 (range77–121)	−1.01/68
0.32
Edinburgh Handedness Inventory	73.29±50.35 (range:−90–100)	55.49±55.58 (range:−100–100)	−1.41/104
0.16
Medication: Mean Fluoxetine equivalent dose (mg) ^⁎^ duration (month)	–	26 patients were on medication 19.52^⁎^2.43	–
State-Trait-Anxiety Inventory-State	28.92±6.43 (range:20–44)	47.70±10.49 (range:29–77)	10.73/62.14 <1.00^⁎^10^−6^
State-Trait-Anxiety Inventory-Trait	31.13±6.78 (range:23–52)	61.44±7.41 (range:45–80)	17.96/104 <1.00^⁎^10^−6^
Short Mood and Feelings Questionnaire	2.63±2.02 (range:0–9)	18.11±5.02 (range:3–26)	22.43/94.07 <1.00^⁎^10^−6^

**Table 2:** Demographic and baseline characteristics in the follow-up group.

	Control (± SD)	Depression (±SD)	Between-group difference (*t* value/d.f.) (*p* value)
Number of subjects	20	13	–
Age (years)	15.78±1.51 (range:12.15–17.76)	15.56±1.28 (range:13.66–17.57)	−0.43/31
0.67
Estimated IQ	101.35±11.16 (range:82–120)	105.71±9.83 (range:92–119)	0.92/25
0.37
Edinburgh Handedness Inventory	68.95±54.16 (range:−90–100)	66.92±47.85 (range:−80–100)	−0.11/31
0.91
Duration between baseline and follow-up (day)	254.62±57.38 (range:191–358)	243.15±49.81 (range:193–369)	0.61/31
0.55
Medication: Mean Fluoxetine equivalent dose (mg) ^⁎^ duration (month)	–	Baseline: 6 people 16.25^⁎^2.13 Follow-up: 5 people 20^⁎^11.25	–
State-Trait Anxiety Inventory-State	28.95±6.53 (range:20–44)	51.15±11.09 (range:32–67)	6.52/17.45
5.00^⁎^10^−6^
State-Trait Anxiety Inventory-Trait	31.15±7.34 (range:23–52)	65.54±7.63 (range:55–80)	12.94/31 <1.00^⁎^10^−6^
Short Mood and Feelings Questionnaire	2.70±1.95 (range:0–9)	18.15±4.81 (range:10–25)	11.01/14.59 <1.00^⁎^10^−6^

**Table 3:** fMRI significant results.

Cluster	Cluster size (voxels)	Maximum Z value	Peak MNI coordinates (X, Y, Z in mm)	Location of the Peak
Sad distractor contrast Mean activation Cluster1	1159	3.97	(−12, 8, 46)	Left supplementary motor cortex
Cluster2	1426	3.60	(−68, −34, −4)	Left middle temporal gyrus
Cluster3	2504	5.02	(−58, −48, 36)	Left supramarginal gyrus
Cluster4	2825	4.13	(14, 0, 14)	Right thalamus
Cluster5	3599	4.98	(−38,−58, −46)	Left cerebellum
Cluster6	30079	5.74	(52, −44, 34)	Right supramarginal gyrus
Happy distractor contrast Mean activation Cluster1	660	3.68	(24, 12, 64)	Right superior frontal gyrus
Cluster2	951	3.45	(48,−50, 34)	Right angular gyrus
Happy distractor contrast Mean deactivation Cluster1	609	3.60	(−60,−54,−12)	Left middle temporal gyrus
Cluster2	1016	3.99	(54, 36, 12)	Right frontal pole
Cluster3	1408	3.82	(0,−12, 14)	Thalamus
Cluster4	3996	4.74	(−52, 36, 10)	Left inferior frontal gyrus
Happy distractor contrast Group difference	309	3.68	(−26, 20, −22)	Left orbitofrontal cortex
