# Supplementary material for: Aberrant brain responses to emotionally valent words is normalised after cognitive behavioural therapy in female depressed adolescents
Source: J Affect Disord. 2016 Jan 1;189:54–61. doi: 10.1016/j.jad.2015.09.008 (PMC4650987; doi:10.1016/j.jad.2015.09.008)
Supplement: Supplementary file 1 — Supplementary material [file mmc1.docx]

**Supplementary Material 1**

**Cognitive Behavioural Therapy (CBT)**

CBT emphasizes collaborative empiricism with phases of assessment, psychoeducation, self monitoring of mood and behaviour, behavioural activation, identification of cognitive biases, cognitive restructuring, problem solving and relapse prevention^1^.

CBT in our study differed from adult CBT in three ways: 1. more focus on developing the therapeutic relationship and engaging the young people in treatment; 2. Flexibility for therapists to place more emphasis on the behavioural part of the CBT if they felt that adolescents would struggle with cognitive elements and; 3. additional options to involve parents in therapy and liaise with schools^1^ (Table 2).

**Supplementary Material 2**

General inclusion criteria^1,2^ for the IMPACT and MR-IMPACT trials were: aged 11 through 17 years; diagnosis of current moderate to severe Diagnostic and Statistical Manual of Mental Disorders (DSM-IV) major depressive disorder as determined by patient and parent interviews of Kiddie Schedule for Affective Disorders and Schizophrenia-Present and Lifetime version (K-SADS-PL); and score of 27 or above on the self-report Moods and Feelings Questionnaire (MFQ)^2^. Exclusion criteria of the IMPACT trial^1^ included: generalized learning difficulties; pervasive developmental disorder; bipolar I disorder; schizophrenia; eating disorder; pregnancy; and currently taking another medication that may interact with an SSRI. Additional exclusion criteria of the MR-IMPACT trial^2^ for all participants (i.e. including controls) were: alcohol dependence; drug dependence; MRI contraindication; brain abnormality; and intolerance to the MRI environment.

Ninety-four female patients and 29 female controls were enrolled, although a number of participants were subsequently excluded from the analysis, for the following reasons: brain abnormality (1 patient), braces (7 patients and 2 controls), schizophrenia comorbidity (1 patient), extreme (above mean+3 standard deviation) motion during scanning (1 patient and 2 control), extreme (above mean+3 standard deviation) behavioural performance (2 patients and 1 control).

The study was conducted in accordance with the Declaration of Helsinki^2^. Ethical approval was granted by the Cambridgeshire 2 Research Ethics Committee. Addenbrooke’s Hospital, Cambridge, UK (REC reference: 09/H0308/168)^2^.

**Supplementary Material 3**

**FMRI data acquisition**

Participants were scanned on a 3T Magnetom Trio Tim MRI scanner (Siemens, Surrey, England) at the Wolfson Brain Imaging Centre, University of Cambridge, UK. Thirty-two (32) slices of data parallel to the anterior-posterior commissure comprised each three-dimensional volume acquired. Acquisition parameters were: echo time, TE=30 ms; repetition time, TR=2 s; flip angle=78°; field of view = 192x120 mm^2^; and 3.0x3.0x3.0 mm^3^ voxel size with an interleaved acquisition^2^.

**Within-subject FMRI data preprocessing**

Imaging data were preprocessed with FEAT (FMRI Expert Analysis Tool) Version 6.00 in FSL (FMRIB’s Software Library, [www.fmrib.ox.ac.uk/fsl](http://www.fmrib.ox.ac.uk/fsl)). Participant head motion was corrected with MCFLIRT^3^, interleaved slice-timing was corrected with Fourier-space time-series phase-shifting, non-brain tissue was removed by BET^4^, spatial smoothing was performed with Gaussian kernel of FWHM 6 mm, grand-mean intensity of data was normalized by a single multiplicative factor, and high-pass temporal filtering with 90 s was performed.

**Supplementary Material 4**

**Patterns of brain activation associated with the task**

Combining all the imaging data, mean activations of all participants (*full group*) were found in the following brain regions: anterior cingulate, cerebellum and insula in the “sad distractor contrast”; superior frontal gyrus and supramarginal gyrus in the “happy distractor contrast”. Mean deactivations of the “happy distractor contrast” were found in dorsolateral prefrontal cortex, temporal cortex and orbitofrontal cortex (Figure 1, Table 3). There was no significant mean deactivation of the “sad distractor contrast”.

**References**

1. Goodyer IM, Tsancheva S, Byford S, Dubicka B, Hill J, Kelvin R, et al. Improving mood with psychoanalytic and cognitive therapies (IMPACT): a pragmatic effectiveness superiority trial to investigate whether specialised psychological treatment reduces the risk for relapse in adolescents with moderate to severe unipolar depres. Trials. BioMed Central Ltd; 2011;12(1):175.

2. Hagan CC, Graham JM, Widmer B, Holt RJ, Ooi C, van Nieuwenhuizen AO, et al. Magnetic resonance imaging of a randomized controlled trial investigating predictors of recovery following psychological treatment in adolescents with moderate to severe unipolar depression: study protocol for Magnetic Resonance-Improving Mood with Psycho. BMC Psychiatry. BMC Psychiatry; 2013;13(1):247.

3. Jenkinson M, Bannister P, Brady M, Smith S. Improved optimization for the robust and accurate linear registration and motion correction of brain images. Neuroimage. 2002;17:825–41.

4. Smith SM. Fast robust automated brain extraction. Hum Brain Mapp. 2002;17:143–55.

**Supplementary Table 1. Comparison between the *follow-up group* and those who had not been followed up (*full group* minus the *follow-up group*)**

|  | Depressed patients | | | Healthy controls | | |
| --- | --- | --- | --- | --- | --- | --- |
|  | Follow-up group  (mean/sd) | Full group minus the follow-up group  (mean/sd) | Between-group difference  (*t*/d.f.)  (*p* value) | Follow-up group  (mean/sd) | Full group minus the follow-up group  (mean/sd) | Between-group difference  (*t*/d.f.)  (*p* value) |
| Number | 13 | 69 | - | 20 | 4 | - |
| Age (years) | 15.56  1.28 | 15.75  1.07 | -0.60/80  0.56 | 15.78  1.51 | 16.47  0.61 | -0.88/22  0.39 |
| Estimated IQ | 105.71  9.83 | 96.41  11.93 | 1.84/44  0.06 | 101.35  11.16 | 98.00  10.07 | 0.56/22  0.58 |
| Edinburgh Handedness  Inventory | 66.92  47.85 | 53.33  56.97 | 0.81/80  0.42 | 68.95  54.16 | 95.00  10.00 | -0.94/22  0.36 |
| State-Trait-Anxiety Inventory-State | 51.15  11.09 | 47.04  10.33 | 1.30/80  0.20 | 28.95  6.53 | 28.75  6.85 | 0.04/22  0.97 |
| State-Trait-Anxiety Inventory-Trait | 65.54  7.63 | 60.67  7.16 | 2.23/80  0.03* | 31.15  7.34 | 31.00  3.37 | 0.06/22  0.96 |
| SMFQ | 18.15  4.81 | 18.10  5.09 | 0.03/80  0.97 | 2.70  1.95 | 2.25  2.63 | 0.40/22  0.69 |
| Happy distractor:  commission | 3.00  3.74 | 1.42  2.81 | 1.76/80  0.08 | 1.75  2.69 | 0.25  0.96 | 1.08/22  0.29 |
| Happy distractor:  omission | -0.69  1.65 | -0.43  3.04 | -0.30/80  0.77 | -0.90  2.47 | -1.50  1.91 | 0.46  0.65 |
| Happy distractor:  reaction time | -23.91  49.44 | -10.51  52.87 | -0.85/80  0.40 | -2.78  41.68 | -31.95  40.81 | 1.28/22  0.21 |
| Sad distractor:  commission | 1.62  1.39 | 0.78  3.43 | 1.48/45.11  0.15 | 0.30  4.49 | 2.50  4.51 | -0.90/22  0.38 |
| Sad distractor:  omission | -0.85  2.82 | 0.59  3.63 | -1.35/80  0.18 | 0.20  2.69 | 1.00  5.60 | -0.45/22  0.66 |
| Sad distractor:  reaction time | 14.89  57.43 | 21.64  41.01 | -0.51/80  0.61 | 36.16  45.34 | 22.13  38.77 | 0.54/22  0.60 |

**Supplementary Table 2. Behavioural ANCOVA results controlling for baseline age**

| Contrast | Behavioural measure | Baseline (mean/sd)  Patient  Control | Baseline group effect (F/*p*) | Group x time interaction (F/*p*) |
| --- | --- | --- | --- | --- |
| Happy distractor | Commission | 1.67±3.01  1.50±2.54 | 0.04  0.85 | 0.23  0.64 |
|  | Omission | -0.48±2.86  -1.00±2.36 | 0.68*  0.41* | 1.12*  0.30* |
|  | Reaction time (s) | -12.63±52.28  -7.64±42.14 | 0.30  0.59 | 0.41  0.53 |
| Sad distractor | Commission | 0.91±3.21  0.67±4.47 | 0.11  0.74 | 0.74  0.40 |
|  | Omission | 0.37±3.54  0.33±3.19 | 0.01  0.94 | 2.78*  0.11* |
|  | Reaction time (s) | 20.57±43.66  32.99±43.80 | 1.25  0.27 | 0.08  0.78 |

*Violations of the ANCOVA were found in these tests, however significant type I errors were not expected from these violations
